# Cullin-4 regulates Wingless and JNK signaling-mediated cell death in the *Drosophila* eye

**DOI:** 10.1038/cddis.2016.338

**Published:** 2016-12-29

**Authors:** Meghana Tare, Ankita Sarkar, Shimpi Bedi, Madhuri Kango-Singh, Amit Singh

**Affiliations:** 1 Department of Biology, University of Dayton, Dayton, OH, USA; 2 Premedical Program, University of Dayton, Dayton, OH, USA; 3 Center for Tissue Regeneration and Engineering at Dayton (TREND), University of Dayton, Dayton, OH, USA; 4 Center for Genomic Advocacy (TCGA), Indiana State University, Terre Haute, IN, USA

## Abstract

In all multicellular organisms, the fundamental processes of cell proliferation and cell death are crucial for growth regulation during organogenesis. Strict regulation of cell death is important to maintain tissue homeostasis by affecting processes like regulation of cell number, and elimination of unwanted/unfit cells. The developing *Drosophila* eye is a versatile model to study patterning and growth, where complex signaling pathways regulate growth and cell survival. However, the molecular mechanisms underlying regulation of these processes is not fully understood. In a gain-of-function screen, we found that misexpression of *cullin-4 (cul-4),* an ubiquitin ligase, can rescue reduced eye mutant phenotypes. Previously, *cul-4* has been shown to regulate chromatin remodeling, cell cycle and cell division. Genetic characterization of *cul-4* in the developing eye revealed that loss-of-function of *cul-4* exhibits a reduced eye phenotype. Analysis of twin-spots showed that in comparison with their wild-type counterparts, the *cul-4* loss-of-function clones fail to survive. Here we show that *cul-4* clones are eliminated by induction of cell death due to activation of caspases. Aberrant activation of signaling pathways is known to trigger cell death in the developing eye. We found that Wingless (Wg) and c-Jun-amino-terminal-(NH_2_)-Kinase (JNK) signaling are ectopically induced in *cul-4* mutant clones, and these signals co-localize with the dying cells. Modulating levels of Wg and JNK signaling by using agonists and antagonists of these pathways demonstrated that activation of Wg and JNK signaling enhances *cul-4* mutant phenotype, whereas downregulation of Wg and JNK signaling rescues the *cul-4* mutant phenotypes of reduced eye. Here we present evidences to demonstrate that *cul-4* is involved in restricting Wg signaling and downregulation of JNK signaling-mediated cell death during early eye development. Overall, our studies provide insights into a novel role of *cul-4* in promoting cell survival in the developing *Drosophila* eye.

During organogenesis, regulation of conserved processes like cell proliferation, cell survival and cell death is crucial for organ growth and differentiation. A fine balance between control of cell death and cell survival is responsible for final organ shape and size during development. We used *Drosophila* eye model to identify genes involved in promoting growth and cell survival. The *Drosophila* adult eye contains 750–800 differentiated ommatidia, and develops from a sac-like epithelial structure called the imaginal disc housed in the larva. The ommatidia differentiate in the wake of a synchronous wave of retinal differentiation called the Morphogenetic Furrow (MF).^1^ The MF originates at the posterior eye margin, and the Wingless (Wg) signaling pathway negatively regulates the anterior movement of the MF.^2, 3, 4^ Wg, a secreted morphogen, initiates an intracellular signaling cascade by binding to its receptors Arrow (Arr) and Frizzled (Fz), which triggers downstream events to control the nuclear localization of the *Drosophila* beta-catenin Arm, and the spatial expression of Wg target genes.^5, 6, 7^ In *Drosophila* eye, Wg is also known to induce the proapoptotic genes, *head involution defective* (*hid*), *reaper* (*rpr*) and *grim* (together referred as HRG), to trigger programmed cell death to remove extra cells from the periphery of the pupal retina.^8, 9, 10, 11, 12, 13^ Further, aberrant signaling during development, *e.g.*, abnormal Wg signaling, also causes stress-induced apoptosis.^14^

The intrinsic caspase-dependent cell death involves activation of HRG,^15, 16, 17^ which are negatively regulated by *Drosophila* inhibitor of apoptosis (DIAPs).^18, 19^ Inactivation of DIAP-1 can trigger cell death by the activation of cysteine proteases Dronc and Drice, the *Drosophila* homolog of Caspase-9 and Caspase-3, respectively.^20, 21, 22, 23^ In *Drosophila* expression of baculo-virus protein, P35 can block caspase-dependent cell death.^24^ Besides Wg, activation of c-Jun amino-terminal (NH_2_) Kinase (JNK) signaling triggers cell death through the activation of caspases.^11, 14, 25, 26, 27^ JNK belongs to a conserved MAP kinase super-family, which is involved in cell proliferation and cell survival, and is activated through a cascade of phosphorylation by MAP kinases.^26, 28, 29, 30^ In *Drosophila*, JNK signaling is activated by binding of the tumor necrosis factor (TNF) Eiger (Egr) to its receptor Wengen (Wgn), and a conserved signaling cascade of Tak 1 (TGFβ activating kinase 1, a Jun kinase kinase kinase (JNKKK), *hemipterous (hep)* (Jun kinase kinase), *basket (bsk)* (Jun kinase) and *jun*. Activation of the pathway leads to expression of the downstream target *puckered (puc)*, a dual phosphatase, which participates in a negative feedback loop by downregulating JNK activity.^29, 30^

We argued that during early eye development, Wg or JNK levels must be tightly regulated to allow differentiation to proceed, and to prevent premature cell death that results in small or reduced eye phenotype. In a genetic screen, we identified *cullin4 (cul-4)* as a modifier that rescues the reduced eye phenotype.^31^ During development *cul-4* is globally required. Analysis of *cul-4* function revealed its new role in promoting cell survival during early eye development. The *cul-4* gene belongs to an evolutionary conserved class of Cullin-family E3 ubiquitin ligases.^32^ Earlier studies showed that *cul-4* is involved in maintenance of genomic integrity by promoting the ubiquitylation and subsequent degradation of key regulators of cell cycle progression.^33, 34, 35, 36^ Here, we report that *cul-4* promotes cell survival by preventing Wg and JNK signaling-mediated cell death in the developing eye.

## Results

### Gain-of-function of *cul-4* rescues reduced eye mutant phenotype

**Figure 1:**
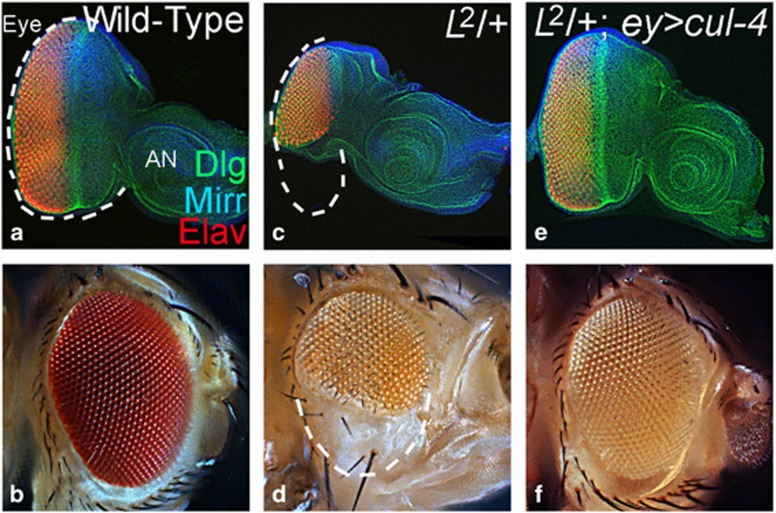
Gain-of-function of *cul-4* can rescue reduced eye mutant phenotype. (**a** and **b**) Wild-type (**a**) eye-antennal imaginal disc and (**b**) adult eye. The eye disc is stained for a membrane specific marker, Dlg (green); dorsal eye fate marker, Mirr (blue); and pan-neural marker, Elav (red). The white dotted line marks the boundary of eye field. (**c** and **d**) *L*^*2*^ mutant exhibits reduced eye phenotype in (**c**) eye imaginal disc and (**d**) adult eye. (**e** and **f**) Misexpression of *cul-4* in the *L*^*2*^ mutant eye background (*L*^*2*^*; ey> cul-4*) results in a significant rescue to a near wild-type eye as seen in (**e**) the eye disc and (**f**) the adult eye

In comparison with the wild-type larval eye disc and the adult eye (Figures 1a and b), *L* mutant exhibits reduced eye phenotype in larval eye disc (Figure 1c) and adults (Figure 1d).^11, 37^ Misexpression of *cul-4* using Gal4/UAS approach^38^ (*L*^*2*^*; ey>cul-4*) resulted in the rescue of *L*^*2*^-reduced eye phenotype (Figures 1e and f). Misexpression of *cul-4* (*ey>cul-4*) does not affect the eye size (not shown) suggesting that *cul-4* may not promote cell proliferation. We analyzed loss-of-function phenotype of *cul-4* to understand its role during eye development.

### Loss-of-function of *cul-4* fail to survive and exhibit reduced eye phenotype

**Figure 2:**
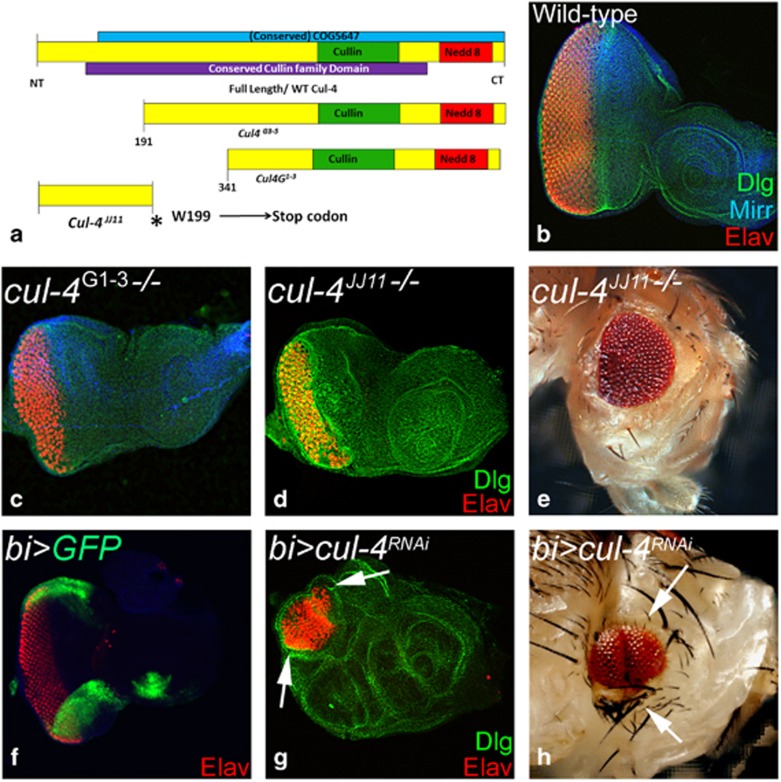
Loss-of-function of *cul-4* results in reduced eye phenotype. (**a**) Schematic representation of wild-type and truncated Cul-4 protein encoded by different mutant alleles of *cul-4* used in the loss-of-function studies.^40^ (**b**) Wild-type eye disc stained for dorsal fate marker Mirr (blue), Dlg (green) and Elav (red). (**c–e**) Loss-of-function clones of *cul-4* (**e**) *cul-4*^*G1−3*^ (**d** and **e**) *cul-4*^*JJ11*^ generated by using 'cell-lethal' approach^39^ results in reduced eye phenotype as seen in (**c** and **d**) eye imaginal disc and (**e**) adult eye. (**f**) *bi*-Gal4 drives expression of GFP reporter on DV of the eye imaginal disc. (**g** and **h**) Misexpression of *cul-4* ^RNAi^ on DV margins of the eye using *bi*-Gal4 driver (*bi>cul4*^*RNAi*^) exhibits eye fate suppression on both DV margins as seen in (**h**) eye disc and the (**i**) adult eye

**Figure 3:**
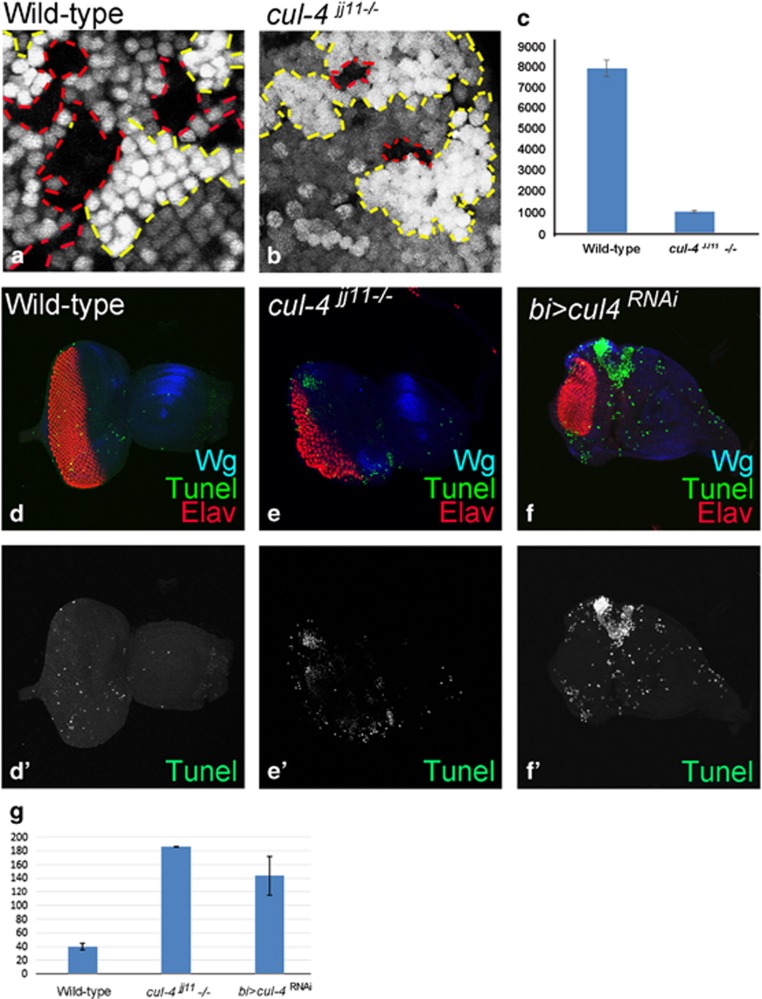
Loss-of-function clones of *cul-4* fail to survive. (**a** and **b**) Genetic mosaics generated by using a Flp-FRT system in the eye results in GFP-positive and GFP-negative patches of cells. (**a**) Note that in wild-type controls, clonal areas marked by the absence of GFP (red dotted lines) are comparable in terms of size to the wild-type clones (with strong (2X) GFP-positive areas, marked by red dotted lines). (**b**) Loss-of-function clones of *cul-4*^*JJ11*^ mutant in the eye imaginal disc (no GFP, marked by red dotted line) are smaller compared with the wild-type twin spot (2XGFP, marked by yellow dotted line). Note that these *cul-4*^*JJ11*^ mutant clones fail to survive 24  h after they are formed. Only GFP-positive (wild-type) cells were seen. (**c**)The cell number of *cul-4* clones was less than eightfold as compared with the wild-type clones based on counting five eye discs for each. (**d–f**') TUNEL staining was used to mark the dying cells nuclei in (**d** and **d**') wild-type, (**e** and **e**') *cul-4*^*jj11*^−/− clones and (**f** and **f**') *bi>cul4*^*RNAi*^ eye imaginal disc. (**g**) The dying nuclei were counted from five imaginal discs from each of these category. There is more than fourfold increase in dying cell population in *cul-4* mutant eye disc as compared with wild-type

We generated *cul-4* loss-of-function clones by *cell lethal (cl)* approach, which results in an eye disc comprising of nearly 80% mutant cells due to elimination of the wild-type twin spot by a *cl* mutation.^39^ Loss-of-function clones of *cul-4* alleles (Figure 2a)^40^ like *cul-4*^ExG1−3^ (Figure 2c) or *cul-4*^*JJ11*^ (Figures 2d and e) resulted in a small eye phenotype as compared with the wild-type eye (Figure 2b). It is to be noted that both *cul-4*^*JJ11*^ and *cul-4*^ExG1−3^ loss-of-function phenotypes were similar in the eye. Downregulation of *cul-4* expression in the dorso-ventral (DV) margins of developing eye disc by using *bi*-Gal4 driver (Figure 2f; *bi>*GFP) resulted in reduction of eye field on DV margins (Figures 2g and h, arrows).^41, 42^ It suggests that there is no domain constraint in *cul-4* function in the eye. It is possible that reduced eye phenotype may be due to induction of cell death. To test this, we used *ey*-Flippase (*ey*-Flp) to induce somatic ‘twin clones' using Ubi-GFP (1XGFP), where *cul-4*^−/−^ mutant cells (GFP-negative) were adjacent to their wild-type twin spot (2XGFP). However, we found that only the wild-type twin clone (2XGFP) and the heterozygous cells (express 1XGFP) could be identified. However, we found wild-type twin clones (2XGFP) but no *cul-4* mutant clones (GFP-negative) in the third-instar eye disc (not shown), suggesting that the *cul-4* mutant cells failed to survive. We performed a 'twin spot' analyses in the heat-shock-Flippase (*hs*-FLP) -induced clones, to test survival profiles of *cul-4* mutant cells. The heat shock was administered in the first instar larva and the resultant clones were examined in the second- (24 h after clone formation (ACF)) and third-instar (48–72 h after clone formation) disc to determine whether or not these clones could survive. Very small clones were detected at second instar stage (within 24 h after clone formation). However, within 48 h after clone formation, the mutant clones were lost. In comparison with the wild-type clones (Figure 3a), the mutant clones generated at later time points (second instar (48 h) or early third instar (60 h)) and examined at late third-instar stage (within 24 h after clone formation), showed smaller *cul-4*^−/−^ clones (Figures 3b and c; clone boundary marked by red dotted lines). These *cul-4*^−/−^ clones failed to survive until 120 h of development. Quantification of clone size showed that *cul-4* mutant clones are significantly smaller than their wild-type twin clones (Figure 3c), suggesting that *cul-4* mutant clones either fail to survive or are slow growing compared with wild-type cells. We tested if *cul-4* mutant cells are eliminated by cell death using TUNEL labeling.^11, 17^ Wild-type eye disc showed few TUNEL-positive dying cells (Figures 3d and d'), whereas eye disc lacking *cul-4* function in the entire eye disc (*cul-4*^*jj11*^*-/-*, Figures 3e and e') or on DV margins (*bi>cul4*^RNAi^, Figures 3f and f') showed a threefold increase in TUNEL-positive cells suggesting that *cul-4* mutant cells are eliminated by cell death.

### *cul-4* prevents cell death in the developing eye

**Figure 4:**
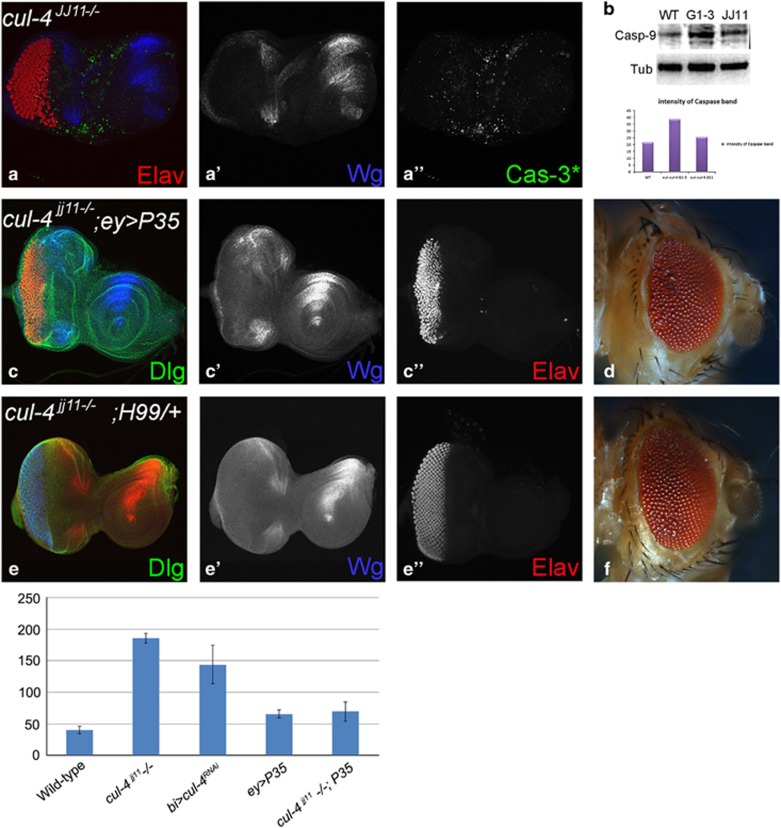
*cul-4* mutant cells are eliminated by activation of caspases. (**a**, **a'** and **a''**) Loss-of-function of *cul-4* in eye results in enhanced caspase-3* (cas-3*, green) levels along with Wg (blue) upregulation and suppression of eye fate based on Elav (red) expression. (**b**) In comparison with the wild-type controls, nearly twofold increase in activator caspase-9 protein are seen in total protein extracted from eye imaginal discs *of cul-4* loss-of-function background. Caspase band staining intensity calculated by Image –J. (**c** and **d**) Loss-of-function phenotype of *cul-4* can be rescued by misexpression of baculo-virus protein, P35 (*cul-4*^−/−^*; ey>P35*) as seen in (**c**, **c'** and **c”**) eye imaginal disc and (**d**) adult eye. (**e** and **f**) Reduction in the levels of Hid-Reaper-Grim (HRG) complex by using deficiency of H99 can rescue the loss-of-function phenotype of *cul-4*, as seen in (**e**, **e'** and **e”**) eye imaginal discs and (**f**) adult eye. (**g**) The dying nuclei were counted from five imaginal discs from each of these category. Misexpression of P35 can significantly reduce the number of dying cell nuclei in *cul-4* loss-of-function eye disc as compared with wild-type

We, therefore, tested if *Drosophila* Caspases- Dronc and Drice activation is part of the mechanism. In the eye disc, *cul-4*^*JJ11*^loss-of-function clones generated by 'cell-lethal' clonal approach,^39^ exhibited robust induction of activated Caspase-3* (Cas-3*) and a signaling molecule Wg (Figures 4a and a''). Similarly, in semi-quantitative western blots, Dronc levels were nearly two fold higher in *cul-4* mutant as compared with the wild-type eye disc (Figure 4b). Thus, both Dronc and Drice are induced in *cul-4* mutant cells. Misexpression of baculo-virus P35 in the *cul-4* loss-of-function clones (*cul-4*^JJ11−/−^, *ey>P35)* resulted in a significant rescue of the reduced eye phenotype (Figures 4c and c”). In *cul-4* loss-of-function background reducing level of proapoptotic genes using H99 deficiency^43^ *(cul-4*^JJ11−/−^, *H99*^*−*^*/+)* resulted in significant rescue of the *cul-*4 mutant phenotype (Figures 4e and e”). It suggests that activation of caspases triggers apoptosis in *cul-4* mutant cells. Aberrant signaling from key developmental and signaling pathways, like Wg/Wnt, can induce apoptosis to prevent defective development.^10, 14^

### *cul-4* represses Wg levels in the developing eye

**Figure 5:**
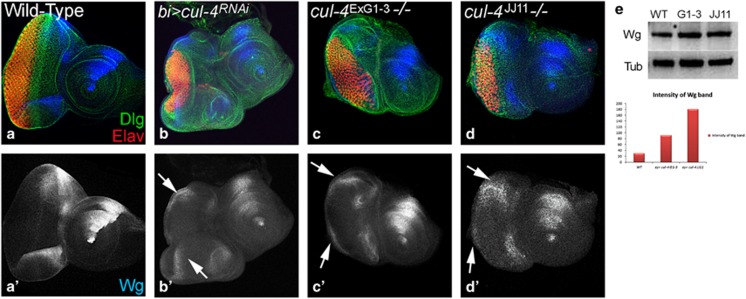
Wg is ectopically induced in *cul-4* mutant background. Expression of Wg (blue) in (**a**,**a**') Wild-type, (**b** and **b'**) *bi>cul4*^*RNAi*^, (*cul-4* ^RNAi^ is misexpressed on DV margin using *bi-*Gal4), (**c** and **c'**) *cul-4*^ExG1−3^and (**d** and **d'**) *cul-4*^*JJ11*^ loss-of-function clones. Note robust ectopic Wg (blue) expression on (**b'**) DV margin (marked by white arrows) along with suppression of eye fate. (**c** and **d**) The reduced eye phenotype of *cul-4* loss-of-function clones generated by *cell-lethal* approach is accompanied by ectopic upregulation of Wg (blue, marked by white arrow). (**a**'–**d**') Shown is the split channel of Wg expression. (**e**) In western blot analysis, the Wg protein levels are more than twofolds in eye discs with *cul-4* loss-of-function clones as compared with wild-type eye disc. Wg band staining intensity calculated by Image-J

In the third-instar stage, Wg is expressed along the antero-lateral margins of the wild-type eye disc (Figures 5a and a'). Reducing *cul-4* function on DV margin of eye disc using *bi*-Gal4 driver (*bi>cul-4*^RNAi^) resulted in strong ectopic induction of Wg in DV domain of the eye (Figures 5b and b'' white arrows). Loss-of-function clones of *cul-4* using the *cul-4*^ExG1−3^(Figures 5c and c') *and cul-4*^*JJ11*^(Figures 5d and d') alleles showed a robust ectopic induction of Wg in the eye disc (Figures 5c', white arrows). Wg levels were significantly upregulated in semi-quantitative western blots on total protein isolated from eye imaginal discs from wild-type, and *cul-4*^ExG1−3^and *cul-4*^*JJ11*^ (Figure 5e). These data suggest that *cul-4* may downregulate Wg in the eye imaginal disc. Misexpression of *wg* on DV margins of eye disc *bi>wg* results in ectopic *wg* transcription suggesting that *wg* is a target of Wg pathway in developing eye (Supplementary Fig. S1). We then tested if aberrant Wg signaling is responsible for *cul-4* mutant phenotypes.

### Wg signaling pathway alters the *cul-4* mutant phenotype

**Figure 6:**
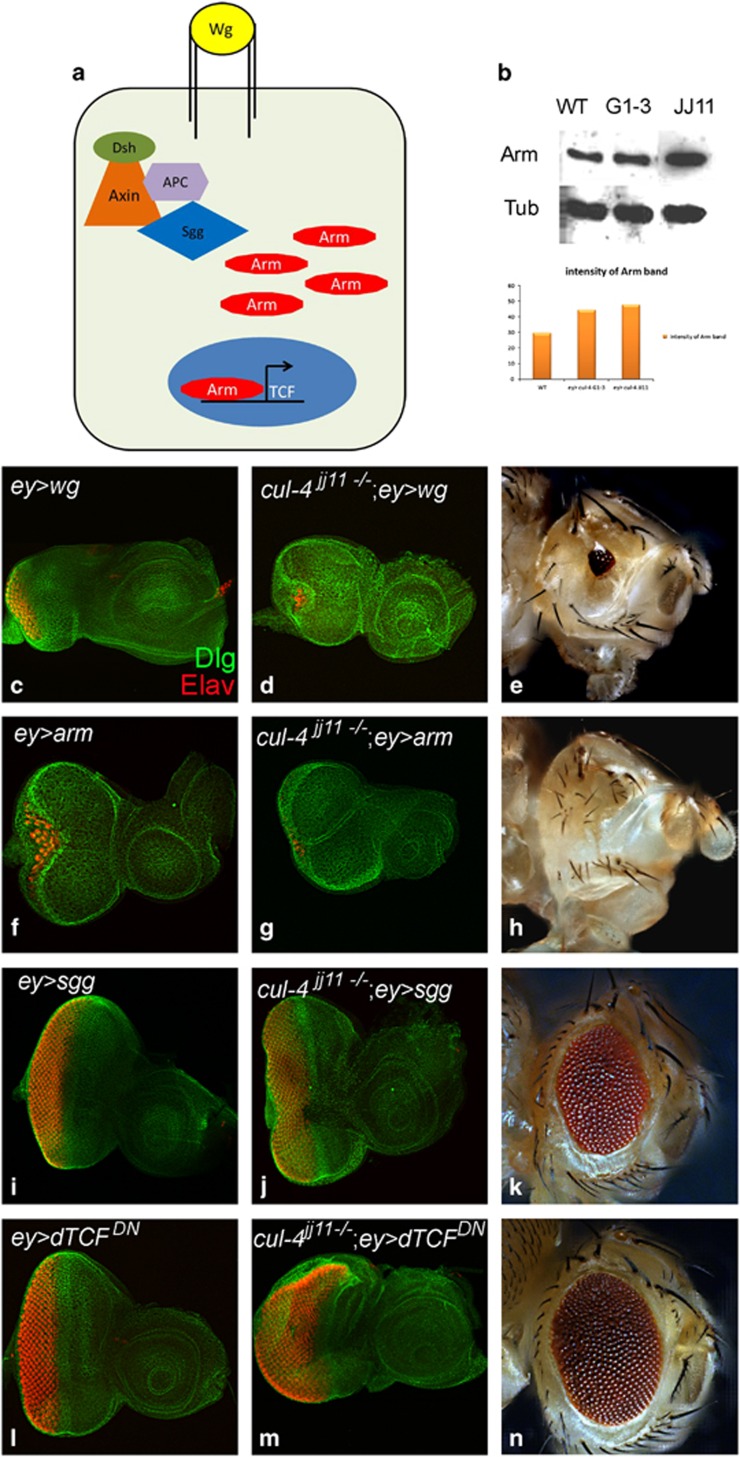
Activation of Wg pathway results in the *cul-4* mutant's reduced eye phenotype. (**a**) Cartoon showing Wg signaling pathway. (**b**) In the western blot performed by using protein extracts prepared from the wild-type and *cul-4* mutant eye imaginal discs, the Armadillo (Arm) (a downstream cytoplasmic target of Wg signaling), protein levels are enhanced (twofold) in *cul-4* loss-of-function background as compared with the wild-type (WT) control. Activation of Wg signaling by misexpression of (**d** and **e**) *wg (cul-4*^*−*/*−*^*; ey>wg)*, (**g** and **h**) *arm* (*cul-4*^*−*/*−*^*; ey>arm*) results in enhancement of loss-of-function phenotype of *cul-4* as seen in (**d** and **g**) eye disc as well as (**e** and **h**) adult eye. (**c** and **f**) Misexpression of (**c**) *wg (ey>wg)* and (**f**) *arm (ey>arm)* results in small eye. Blocking Wg signaling by misexpression of negative regulators/ antagonists like (**j** and **k**) *sgg*^S9A^ (*cul-4*^*−*/*−*^*; ey> sgg*^S9A^) and (**m** and **n**) *dTCF*^*DN*^(*cul-4*^*−*/*−*^*; ey> dTCF*^*DN*^) suppresses the reduced eye phenotype of *cul-4* loss-of-function to near normal as seen in (**j** and **m**) eye disc and (**k** and **n**) adult eye. Misexpression of (**i**) *sgg (ey>sgg*^S9A^) and (**l**) *dTCF*^*DN*^*(ey>dTCF*^*DN*^) results in normal eye sizes

Arm translocates to the nucleus in response to Wg signaling and binds with the transcription factor dTCF (LET/TCF family protein) to turn on the transcription of Wg target genes (Figure 6a).^44^ In western blots a two fold increase in Arm protein levels was observed in *cul-4* mutant eye discs as compared with wild-type (Figure 6b). We tested if modulating Wg signaling levels can affect the *cul-4* mutant phenotype. In the eye imaginal discs, activation of Wg signaling by misexpression of *wg* (*ey>wg*) (Figure 6c) or *arm* (*ey>arm*) (Figure 6f) resulted in reduced eye phenotypes.^11^ Misexpression of *wg* (*cul-4*^JJ11−/−^*, ey>wg*) (Figures 6d and e) or *arm* (*cul-4*^*JJ11*^*, ey>arm*) (Figures 6g and h) in *cul-4* loss-of-function background, resulted in near complete loss of eye. Blocking Wg signaling by misexpression of a constitutive active form of Shaggy/Zeste-White-3/GSK-3 (Sgg), a negative regulator of the Wg signaling pathway^4, 45^ (*ey>sgg*^*S9A*^) (Figure 6i),^11^ or dominant negative form of TCF (*dTCF*^*DN*^)^44^ (*ey*>*dTCF*^*DN*^) (Figure 6l) does not affect the size of the eye field. However, in *cul-4* loss-of-function background, misexpression of *sgg* (*cul-4*^JJ11−/−^*, ey>sgg*) (Figures 6j and k) or *dTCF*^*DN*^ (*cul-4*^JJ11−/−^*, ey>dTCF*^*DN*^), (Figures 6m and n) resulted in a significant rescue of the *cul-4* loss-of-function phenotype to a near wild-type eye. It suggests that *cul-4* is involved in downregulation of Wg signaling in the eye. JNK is known to work in conjunction with Wg in multiple contexts including correction of morphogen gradient discontinuities;^26^ and differential levels of JNK signaling are associated with cell survival.^26, 28^

### *cul-4* prevents JNK-mediated cell death in the developing eye

**Figure 7:**
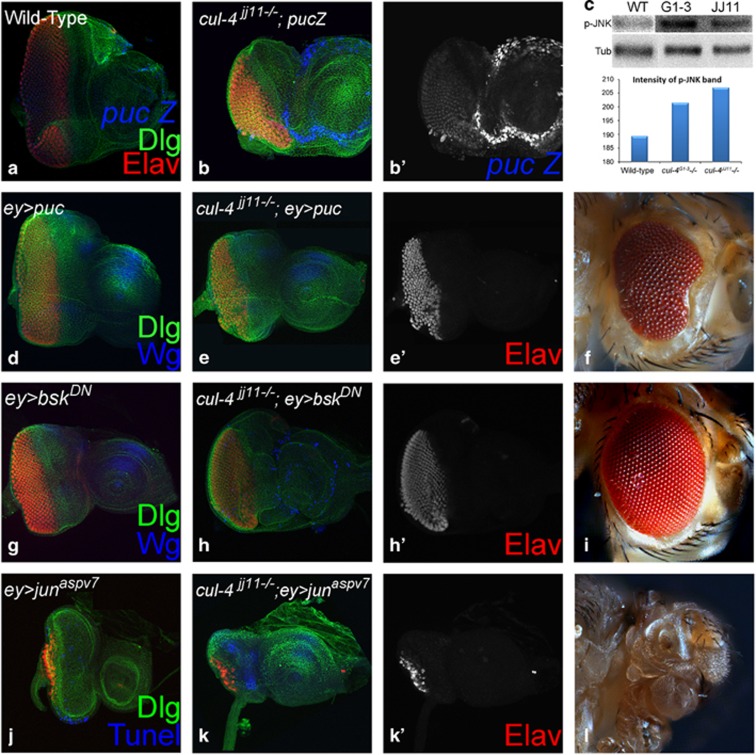
Aberrant JNK signaling in *cul-4* loss-of-function background triggers cell death. (**a**) A functional readout of JNK signaling pathway, *puc-lacZ* (blue) is expressed in differentiated photoreceptor neurons (marked by Elav, red) in the wild-type eye discs.^60^ (**b** and **b**') Loss-of-function of *cul-4* causes ectopic induction of *puc-lacZ* reporter in the eye imaginal disc. (**c**) Activation of JNK signaling was detected by analyzing phospho-Jun (p-JNK) levels in western blots. Twofold increase in the levels of JNK signaling *pathway was detected in cul-4* loss-of-function backgrounds in comparison to the wild-type eye imaginal disc. (**d–i**) Blocking JNK signaling by misexpression of (**e**, **e'** and **f**) *puc* (*cul-4*^*−*/*−*^*; ey>puc)* or (**h**, **h'** and **i**) dominant negative *bsk*^*DN*^(*cul-4*^*−*/*−*^; ey>bsk^*DN*^) in loss-of-function clones of *cul-4* significantly restores their reduced eye phenotype as seen in the (**e**, **e**', **h** and **h**') eye imaginal disc and the (**f** and **i**) adult flies. Misexpression of (**d**) *puc* (*ey>puc*) or (**g**) *bsk* dominant negative (*ey>bsk*^*DN*^) does not affect the eye size. (**j–l**) Misexpression of activated form of (**j**) *jun* (*ey>jun*^*aspv7*^) in the developing eye results in reduced eye size, whereas (**k**, **k'** and **l**) misexpression of activated *jun* (*cul-4*^*−*/*−*^; *ey>jun*^*aspv7*^) results in further enhancement of *cul-4* loss-of-function phenotype of reduced eye

We tested if JNK pathway is associated with the *cul-4* loss-of-function phenotypes (Figure 7). We tested JNK levels in *cul-4* loss-of-function background using *puc (puc-lacZ)* the downstream target, which serves as the functional readout for JNK pathway activation.^30^ In wild-type eye disc, *puc* is expressed in differentiated photoreceptor neurons of eye disc (Figure 7a). In *cul-4* loss-of-function background, ectopic induction of *puc* was seen in the eye as well antenna disc (Figures 7b and b'), suggesting that JNK signaling is activated in *cul-4* mutant cells. To confirm, we checked levels of p-JNK, a reporter for activated JNK signaling, in western blots and found significant upregulation of p-JNK levels in *cul-4* mutants compared with wild-type eye disc (Figure 7c). Blocking JNK signaling in the developing eye by misexpression of *puc (ey>puc,* Figure 7d) or *bsk* dominant negative (*bsk*^*DN*^) (*ey>bsk*^*DN*^, Figure 7g)^25^ did not affect the eye size. However, in *cul-4* loss-of-function background misexpression of *puc* (*cul-4*^JJ11−/−^*, ey>puc*, Figures 7e and e') or *bsk*^*DN*^ (*cul-4*^JJ11−/−^*, ey> bsk*^*DN*^, Figures 7h and h'), resulted in a significant rescue of *cul-4* loss-of-function phenotype of reduced eye (Figure 2). Conversely, in *cul-4* loss-of-function background activation of the JNK signaling pathway by misexpression of activated Jun (*jun*^*aspv7*^) (*cul-4*^JJ11−/−^*, ey>jun*^*aspv7*^) in the eye disc, strongly enhanced the reduced eye- to a 'no-eye' phenotype (Figures 7k and k'). Misexpression of *jun*^*aspv7*^(*ey>jun*^*aspv7*^) alone in the eye results in a highly reduced eye field (Figure 7j). It suggests that loss of *cul-4* leads to activation of JNK signaling in the eye.

**Figure 8:**
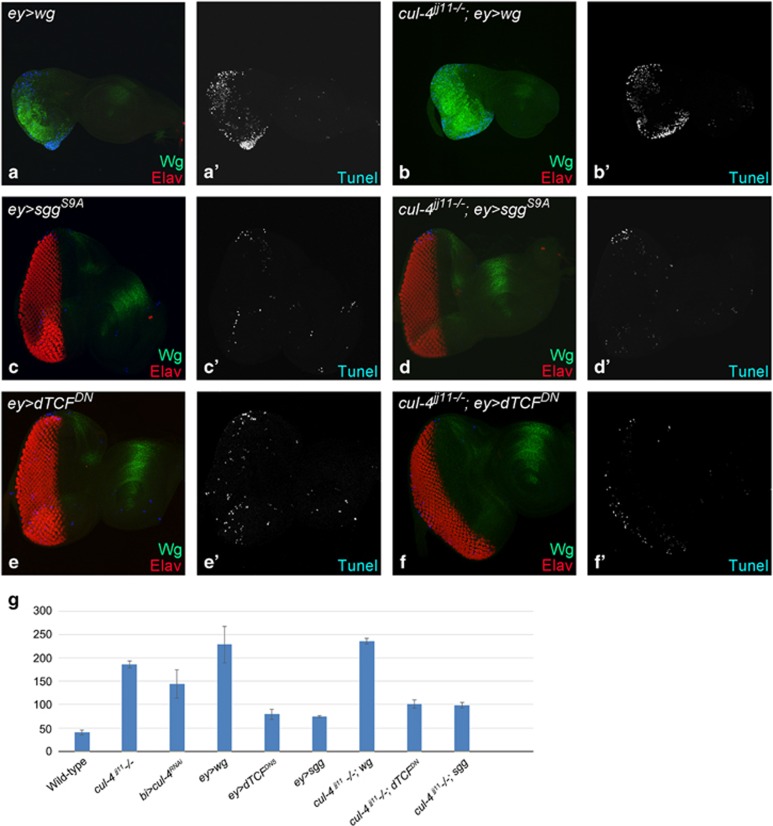
Aberrant Wg signaling triggers cell death in cul-4 loss-of-function background. Eye imaginal discs showing TUNEL (blue) labeling in (**a** and **a**') ey>wg, (**b** and **b**') *cul-4*^−/−^*; ey>wg* (**c** and **c**') *ey>sgg*^*S9A*^, (**d** and **d**') *cul-4*^−/−^*; ey>sgg*^*S9A*^, (**e** and **e**') *ey>dTCF*^*DN*^ and (**f** and **f**') *cul-4*^−/−^*; ey> dTCF*^*DN*^. (**g**) Graph shows quantification of the number of dying cells in the wild-type, cul-4 loss-of-function and genotypes shown in (**a**–**f**). Note that in cul-4 loss-of-function background cell death is enhanced when Wg is activated. Conversely, cell death is suppressed to near wild-type when Wg signaling is blocked in cul-4 loss-of-function background

**Figure 9:**
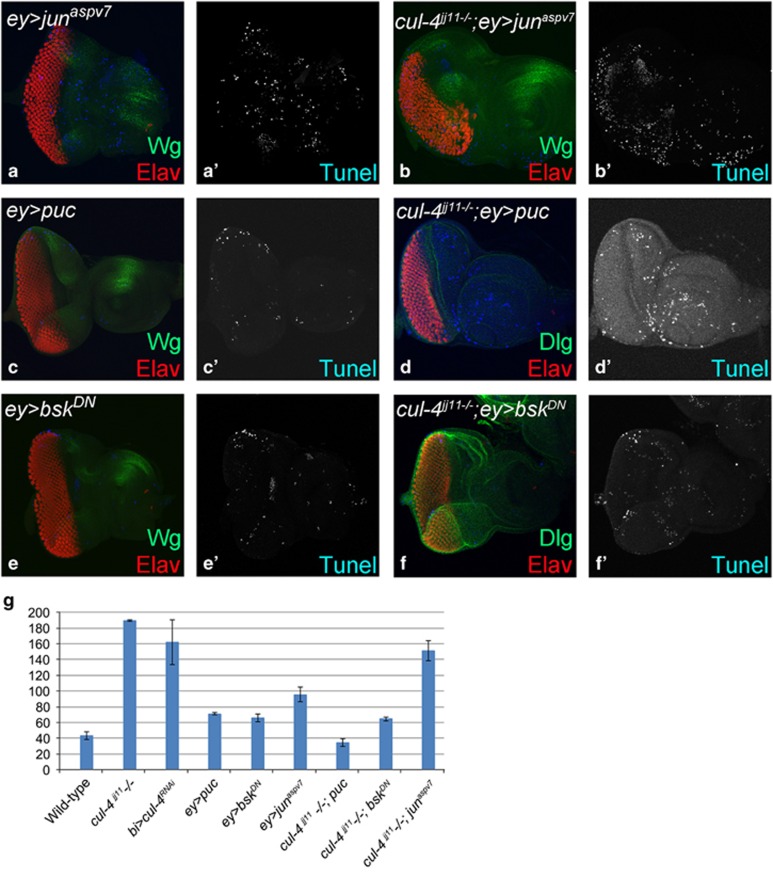
Aberrant JNK signaling triggers cell death in cul-4 loss-of-function background. Panels show TUNEL (blue) staining marking the dying cells nuclei in eye discs from (**a** and **a'**) *ey>jun*^*aspv7*^, (**c** and **c'**) *ey>puc*, (**e** and **e'**) *ey>bsk*^*DN*^, (**b** and **b'**) *cul4*^−/−^*; ey>jun*^*aspv7*^, (**d** and **d'**) *cul4*^−/−^*; ey>puc* or (**f** and **f'**) *cul4*^−/−^; *ey> bsk*^*DN*^. (**g**) Graph shows a comparison of the number of dying cells in wild-type, *cul-4* loss-of-function and the genotypes shown in **a**–**f**. Note that rate of cell death is enhanced when JNK signaling is activated whereas the rate of cell death is suppressed when JNK signaling is blocked in *cul-4* loss-of-function background

To confirm that activation of Wg /JNK signaling pathway are both associated with the induction of cell death observed in *cul-4* mutant cells, we monitored cell death using TUNEL assay when Wg (Figure 8) and JNK (Figure 9) levels are modulated in the wild-type, and in *cul-4* mutant eye discs. We found that cell death is reduced when Wg or JNK signaling is downregulated in *cul-4* mutant background. However, cell death is elevated when Wg/ JNK signaling is activated. Thus, *cul-4* may be involved in limiting JNK as well as Wg activation in the developing eye disc, and thereby promote cell survival during development.

## Discussion

Cul-4, an E3 ligase, is involved in regulation of chromatin function through heterochromatin gene silencing, maintenance of genomic integrity by promoting the ubiquitylation and degradation of key cell cycle regulators.^46, 47, 48, 49^ A number of ligases work in concert with the signaling pathways (Notch (N), Hedgehog (Hh), Wg and so on) for regulating gene expression. For example, Slimb, is involved in regulating Wg and Hh signaling during eye development,^50^ Neuralized (Neu)^51^ and Mind Bomb (Mib), are E3 ligases that are components of N signaling pathway; and are required for *Drosophila* eye development.^52^ Recently other functions for E3 ligases are being recognized. For example, DIAP1 regulates Dronc/Hid caspases,^20, 53^ and is transcriptionally regulated by *yorkie (yki)* for survival function.^54^ DIAP-1 in turn, is regulated by Cul-3 in the developing eye to regulate apoptosis.^71^ Our studies provide evidences for a new function for *cul-4* in cell survival during eye development.

Homozygous larvae of some *cul-4* alleles are larval lethal that can survive until early third instar and produce smaller imaginal discs than wild-type discs at comparable developmental age.^40^ These phenotypes were attributed to problems with cell division. Our twin spot analysis revealed an interesting result that *cul-4* mutant tissues in the developing eye imaginal disc failed to survive (Figure 2), and are eliminated by activation of caspases (Figure 3). Generating *cul-4* mutant clones by using multiple approaches (for example, *eyeless* and *heat-shock* flippase) validated that *cul-4* mutant cells failed to survive when generated in early embryonic or larval stages. Blocking caspase-mediated cell death led to significant rescue of reduced eye phenotypes of *cul-4* loss-of-function (Figures 4c and d), supporting a role for *cul-4* in cell survival.

**Figure 10:**
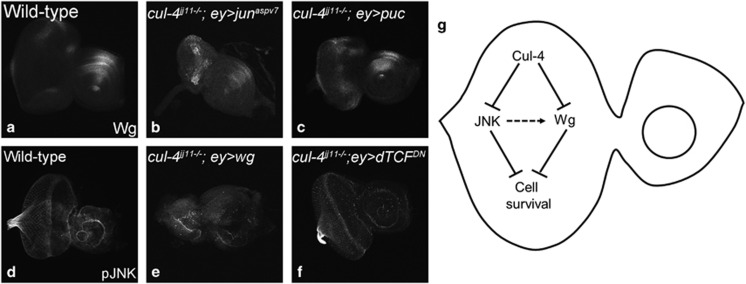
cul-4 promotes cell survival by regulating wg and JNk signaling in the developing eye. (**a**–**c**) Wg expression in (**a**) wild-type, (**b**) *cul-4*^−/−^*; ey> jun*^*aspv7*^; and (**c**) *cul-4*^−/−^*; ey>puc* eye imaginal disc. Note that Wg levels are upregulated when JNK signaling is activated, whereas Wg levels are not much affected when JNK signaling is downregulated. p-JNK expression in (**a**) wild-type, (**b**) *cul-4*^−/−^*; ey> wg*; and (**c**) *cul-4*^−/−^*; ey>dTCF*^*DN*^ eye imaginal disc. Note that pJNK levels are not affected. (**g**) During eye development c*ul-4* is involved in (i) downregulating Wg signaling and (ii) inhibiting JNK signaling to promote cell survival. Activated JNK signaling can trigger Wg induction

We tested several cell signaling pathways in *cul-4* loss-of-function background and found aberrant activation of Wg and JNK signaling (Figure 10). Wg is required for patterning, growth regulation and cell survival in multiple tissues including the eye discs. Ectopic induction of Wg induces cell death.^9, 10, 11, 55^ We found that cells lacking *cul-4* function also undergo cell death and they express high levels of Wg. Arm, the nuclear effector of the Wg signaling pathway, is a target of E3 ubiquitin ligase-mediated degradation.^56^ Loss-of-function phenotype of *cul-4* mutants could be modified by modulating the levels of canonical Wg signaling (Figures 6 and 7). Our data suggests that *cul-4* may downregulate Wg signaling in the eye to promote cell survival in the eye disc. Since the *cul-4* mutant phenotype was not completely rescued by blocking Wg signaling, we also tested the JNK signaling in the *cul-4* mutant clones. The possibility of indirect consequences responsible for the mutant phenotype can be refuted because these phenotypes can be rescued by blocking Wg as well as JNK-mediated cell death. We found that Wg levels were affected when JNK signaling was modulated in cul-4 mutant background (Figure 10a–c). However, the converse did not show effect on phospho-JNK levels (Figure 10d–f). Our studies generate insights into genetic mechanisms that regulate cell survival during normal development by demonstrating the role of *cul-4* in preventing inappropriate upregulation of Wg and JNK signaling in the developing *Drosophila* eye during early stages (Figure 10). A recent study showed that loss of Godzilla, a member of the RNF family of membrane-anchored E3 ubiquitin ligases regulates Wg levels on the basolateral surface of the tissues through dynamin-dependent endocytosis from the apical surface and subsequent trafficking from early apical endosomes to the basolateral surface.^57^ Our studies also generate mechanistic insights into genetic mechanisms that regulate cell survival during normal development.

Numerous studies have shown the role of ubiquitin-mediated proteolysis in a broad array of cellular processes like defects in organogenesis, growth, differentiation, metabolism and aging in all organisms.^58^ Abnormal protein homeostasis underlies various disorders ranging from growth defects to neurodegenerative disorders.^59^ Our studies introduced new role of *cul-4* in cell survival in the developing *Drosophila* eye. Since *Drosophila* serves as an excellent model to study development and human disease,^60^ these studies may shed light on understanding genetic basis of neurodegenerative orders in future.

## Materials and methods

### Fly stocks

The fly stocks used are described on Flybase (http://flybase.bio.indiana.edu). Cul-4 stocks used are EP 2518 (UAS-*cul-4*); *cul-4* RNAi lines 8711 and 8711-R1 (from NIG).^61^ The N-terminal deletion mutants used were *cul-4* ^ExG1−3^*/CyO,* which lack 340 amino acids from amino terminal. c*ul-4*^*JJ11*^*/ twi>GFP, CyO* carries a nonsense mutation at Trp199 position.^40^

Other stocks include Canton-S, *y w ey*FLP,^39^ *L*^*2*^*/Cyo;*^37, 62^ *wg-lacZ/CyO*,^63^ UAS-wg,^64^ UAS-sgg^S9A^,^65^ UAS-*arm*,^66^ UAS*-dTCF*^*DN*^,^44^ UAS*-P35*,^24^ Df(3L)H99/TM6B,^43^ *puc*^*E69*^, UAS-*puc*,^30^ UAS*-bsk*^DN^,^25^ and UAS*-DJun^aspv^*^7^.^67^ The Gal4/UAS system was used for targeted misexpression studies^38^ using *ey-*Gal4,^65^ and *bi-*Gal4 (refs 41, 42) lines.

### Mosaic analysis

To generate loss-of-function clones^68^ of cul-4 in the eye, virgins of eyFlp; FRT42D, cl-w^+^/CyO-GFP were crossed to (i) FRT 42D, cul-4 ^ExG1*−*3^/CyO, (ii) FRT 42D, cul-4 ^ExG3*−*5^, (iii) FRT 42D, cul-4 ^ExL2*−*1^/CyO and (iv) FRT 42D, cul-4 ^JJ11^/twi>GFP, CyO.

### Twin spot analysis

We used *hsFlp; FRT42D* ubi-GFP to generate loss-of-function clones of *cul-4* ^ExG1−3^ and *cul-4* ^*JJ11*^in the eye imaginal disc at different larval development stages. Egg laying were collected from synchronous cultures maintained at 25 °C. The cultures were heat shocked at 24 and 48 h after egg laying (AEL) at 37 °C for 50 min in order to induce loss-of-function clones. Eye discs were dissected in second and third-instar stages to analyze/identify *cul-4* loss-of-function clones marked by the absence of GFP expression.

### Immunohistochemistry

Eye-antennal imaginal discs were dissected from wandering third-instar larvae and stained following the standard protocol.^62^ Antibodies used were rat anti-Elav (1:100), mouse anti-Wg (1:50), mouse anti-β galactosidase (1:200) (Developmental Studies Hybridoma Bank, DSHB, Iowa City, IA, USA), rabbit anti-Dlg (1:250) (gift from Kyung- Ok Cho), rat anti-Mirror (1:200) (gift from Kwang Wook Choi), rabbit anti-caspase-3* (1:200) and rabbit Phospho-SAPK/JNK (Cell Signaling Thr183/Tyr185) (81E11) (Cell Signaling Technology, Danvers, MA, USA). Secondary antibodies (Jackson Immuno Research Laboratories Inc., West Grove, PA, USA) were goat anti-rat IgG conjugated with Cy5 (1:200), donkey anti-rabbit IgG conjugated to Cy3 (1:250), donkey anti-rabbit IgG conjugated to FITC and donkey anti-mouse IgG conjugated to Cy3 (1:200). The discs were mounted in Vectashield (Vector Laboratories Inc., Burlingame, CA, USA) and imaged using Olympus Fluoview 1000 microscope (Olympus America, Scientific Solutions Group, Center Valley, PA, USA). Images were analyzed using the Adobe Photoshop CS4 (Adobe Systems, San Jose, CA, USA) and image intensity was calculated using the Image J software.

### TUNEL assays

Apoptotic cell death was assayed using TUNEL assays in the mutant clones generated via twin spot analysis and cell lethal approach. Eye discs, after secondary antibody staining,^62^ were blocked in 10% Normal Goat Serum in Phosphate Buffered Saline with 0.2% Triton X-100.TUNEL assays were done using the Cell-death Detection Kit from Roche Diagnostics following the standardized protocol.^11, 17^ The TUNEL-positive nuclei were counted from five eye imaginal discs for each genotype using Image-J and statistical analysis was performed using Microsoft Excel 2013. The P-values were calculated and the error bars represent Standard Deviation.

### Adult eye imaging

Adult *Drosophila* eye images were taken^70^ using a Zeiss Apotome Imager.Z1 microscope (Carl Zeiss Microscopy GmbH, Jena, Germany). The flies were prepared by freezing them at −20 °C for ~2 h. The legs and wings of the flies were removed and flies were mounted on a dissection needle, and the fly was positioned on a glass slide using mounting putty. Images were captured by using extended depth of focus function of the Axiovision software version 4.6.3 (Carl Zeiss Microscopy GmbH, Jena, Germany) by compiling the individual stacks from the Z-sectioning approach. The final images and figures were prepared using Adobe Photoshop CS4 software.

### Western blot analysis

Protein samples were prepared from third-instar eye-antennal imaginal discs of different *cul-4* mutants dissected in ice-cold PBS. Samples were transferred to sample buffer containing SDS-β-mercaptoethanol, boiled for 10 min, stored in −80 °C. Protein samples were separated on 10% SDS-PAGE and transferred to nitrocellulose membrane. The membrane was blocked in blocking solution (AMRESCO LLC, Solon, OH, USA) and incubated with primary antibody. The antibodies used were anti- mouse Wg (1:100) (DSHB); anti-mouse arm (1:2000) (DSHB), anti-rabbit p-JNK (1:2000) (Cell signaling Technologies), anti-rabbit Caspase-9 (1:1000) (Cell signaling Technologies) or anti- mouse tubulin (1:5000) (Sigma-Aldrich Corp., St. Louis, MO, USA). Secondary antibodies were horseradish peroxidaseconjugated goat anti-rabbit IgG, and the signal was detected using super-signal chemiluminiscence substrate (Pierce Biotechnology, Thermofisher Scientific, Rockford, IL USA).

## Abbreviations

Cul-4: Cullin-4
Wg: Wingless
JNK: c-Jun amino-terminal (NH_2_) Kinase
MF: Morphogenetic Furrow
Arm: Armadillo
dTCF: Drosophila T cell factor
Arr: Arrow
Fz: Frizzled
Sgg: Shaggy
Hid: head involution defective
Rpr: reaper
HRG: hid, reaper, and grim
DIAP1: Drosophila inhibitor of Apoptosis
PCD: programmed cell death
Cas-9: caspase-9
Cas-3: Caspase-3
TNF: Tumor necrosis factor
Egr: Eiger
Wgn: Wengen
Tak1: TGFβ activating kinase 1
Hep: hemipterous
Bsk: basket
Bsk^DN^: basket dominant negative
Puc: puckered
L: Lobe
Ey: eyeless
UAS: Upstream Activator Sequence
Cl: cell lethal
Bi: bifid
DV: Dorso-ventral
GFP: Green Fluorescent Protein
FLP: Flippase
ACF: after clone formation
RNAi: RNA interference
N: Notch
Hh: Hedgehog
Neu: Neuralized
Mib: Mind Bomb
Yki: yorkie
Cul-3: Cullin-3
RNF: Ring finger proteins
NIG: National Institute of Genetics
Twi: twist
FRT: Flippase recombination targets
AEL: after egg laying
Dlg: disc large
β: beta
Elav: embryonic lethal, abnormal vision
IgG: Immunoglobulin G
FITC: Fluorescein isothiocyanate
Cy3: Cyanines 3 dye
Cy5: Cyanines 5 dye
