# Supplementary figures and images for: Cullin-4 regulates Wingless and JNK signaling-mediated cell death in the Drosophila eye
Source: Cell Death Dis. 2016 Dec 29;7(12):e2566–. doi: 10.1038/cddis.2016.338 (PMC5261020; doi:10.1038/cddis.2016.338)

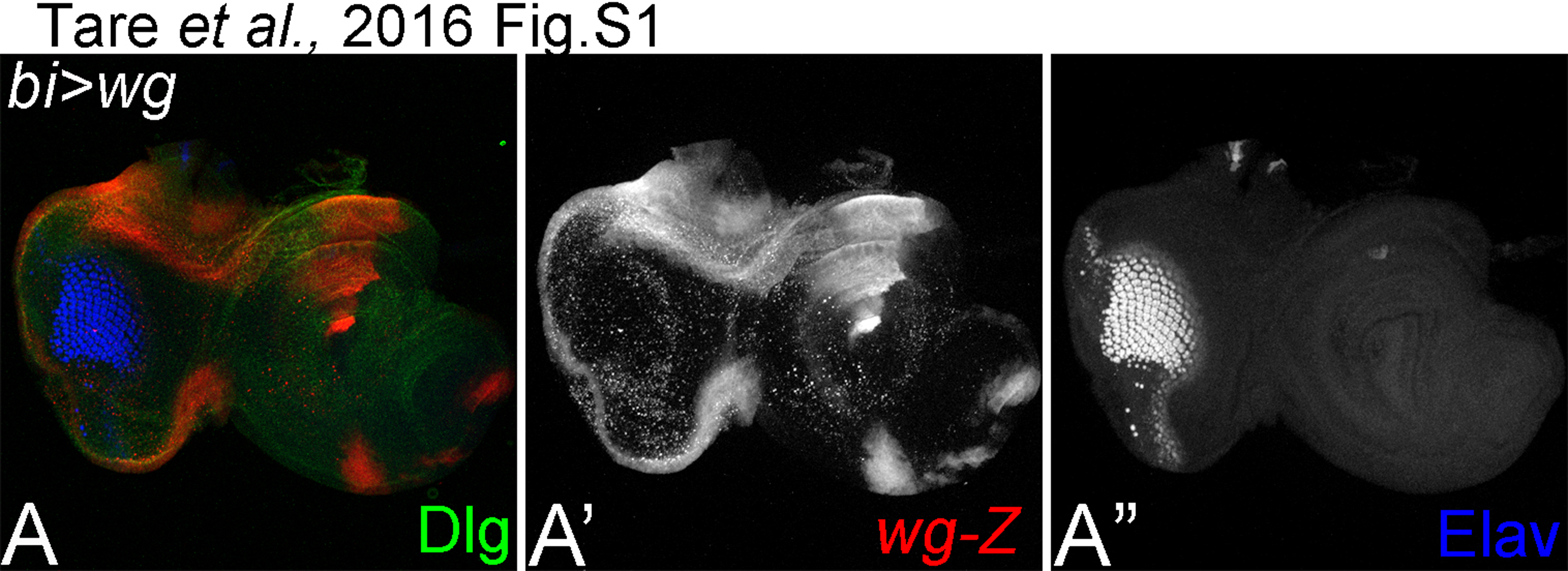

Supplement: Supplementary Figure 1 [file cddis2016338x1.tif]
